# Late-Onset Acute Kidney Injury is a Poor Prognostic Sign for Severe Burn Patients

**DOI:** 10.3389/fsurg.2022.842999

**Published:** 2022-05-02

**Authors:** Bo You, Zichen Yang, Yulong Zhang, Yu Chen, Yali Gong, Yajie Chen, Jing Chen, Lili Yuan, Gaoxing Luo, Yizhi Peng, Zhiqiang Yuan

**Affiliations:** ^1^ Department of Burn and Plastic Surgery, No. 958 Hospital of PLA Army, Third Military Medical University (Army Medical University), Chongqing, China; ^2^ State Key Laboratory of Trauma, Burns and Combined Injury, Institute of Burn Research, Southwest Hospital, Third Military Medical University (Army Medical University), Chongqing, China; ^3^ Department of Plastic and Cosmetic Surgery, Xinqiao Hospital, Third Military Medical University (Army Medical University), Chongqing, China; ^4^ Department of Burn and Plastic Surgery, General Hospital of Xinjiang Military Region, PLA, Xinjiang, China

**Keywords:** acute kidney injury, burn, prognosis, sepsis, risks

## Abstract

**Background:**

Acute kidney injury (AKI) is a morbid complication and the main cause of multiple organ failure and death in severely burned patients. The objective of this study was to explore epidemiology, risk factors, and outcomes of AKI for severely burned patients.

**Methods:**

This retrospective study was performed with prospectively collected data of severely burned patients from the Institute of Burn Research in Southwest Hospital during 2011–2017. AKI was diagnosed according to Kidney Disease Improving Global Outcomes (KDIGO) criteria (2012), and it was divided into early and late AKIs depending on its onset time (within the first 3 days or >3 days post burn). The baseline characteristics, clinical data, and outcomes of the three groups (early AKI, late AKI and non-AKI) were compared using logistic regression analysis. Mortality predictors of patients with AKI were assessed.

**Results:**

A total of 637 adult patients were included in analysis. The incidence of AKI was 36.9% (early AKI 29.4%, late AKI 10.0%). Multiple logistic regression analysis revealed that age, gender, total burn surface area (TBSA), full-thickness burns of TBSA, chronic comorbidities (hypertension or/and diabetes), hypovolemic shock of early burn, and tracheotomy were independent risk factors for both early and late AKIs. However, sepsis was only an independent risk factor for late AKI. Decompression escharotomy was a protective factor for both AKIs. The mortality of patients with AKI was 32.3% (early AKI 25.7%, late AKI 56.3%), and that of patients without AKI was 2.5%. AKI was independently associated with obviously increased mortality of severely burned patients [early AKI, OR = 12.98 (6.08–27.72); late AKI, OR = 34.02 (15.69–73.75)]. Compared with patients with early AKI, patients with late AKI had higher 28-day mortality (34.9% vs. 19.4%, *p* = 0.007), 90-day mortality (57.1% vs. 27.4%, *p* < 0.0001).

**Conclusions:**

AKI remains prevalent and is associated with high mortality in severely burned patients. Late-onset acute kidney injury had greater severity and worse prognosis.

## Background

Severely burned patients often develop acute kidney injury (AKI), which contributes to high morbidity and mortality in these patients (1), even if they arrive at the hospital timely with adequate fluid resuscitation (2, 3). The median mortality of AKI was 34.9% in patients with severe burn injury (4), while that was 80% in the patients in need of renal replacement therapy (RRT) (5). To prevent AKI post-burn, it is crucial to identify high-risk factors so that relevant prophylactic measures can be taken in time (6, 7). Advances have been made in the understanding of AKI pathophysiology in these patients. However, this had not been translated to significant advances in treatment or an improvement in mortality outcome until recently. It is still hard to prognosticate and prevent the occurrence or progression of AKI after severe burn injury. Previous research focusing on AKI mostly reports events after general trauma (5), in which the prevalence and risk factors have been properly addressed. However, the inflammatory response, levels of hormone and inflammatory mediators in burn injury are different from those in other kinds of trauma (8). Compared with general trauma, the pathological differences in severe burn injuries make AKI post burn more difficult to predict (6). Severe burn injury initiates AKI-risk factors including trauma stress (5, 9), hypovolemic shock resulted from fluid and protein translocation in early stage, as well as excess tissue fluid returning to blood (10), invasion of pathogenic microorganisms and sepsis in the late stage (11). Therefore, reducing the incidence of AKI and improving the prognosis of burned patients with AKI are still facing significant difficulties and challenges. Prevention and mitigation nephrotoxicity are best strategies to attenuate AKI risk or progression, although AKI is often unavoidable in severely burned patients. Effective prevention and treatment methods are still being explored. Clinical characteristics, inflammatory response, levels of hormone and inflammatory mediators are also different in different periods post severe burn. Therefore, it is reasonable for AKI be classified into early AKI (0–3 days after injury) or late AKI (more than 3 days after injury) depending on its onset time after burn (1). We hypothesized that AKI is frequently encountered and the time of occurrence of AKI is associated with mortality of severely burned patients.

The aims of the present study were (1) to describe the epidemiology of AKI in a large burn center, (2) to evaluate the risk factors for AKI in severely burned patients, and (3) to assess the relationship between AKI (early and late AKI) and mortality.

## Methods

We conducted a retrospective observational study in the Institute of Burn Research, First Affiliated Hospital (Southwest Hospital) of the Third Military Medical University, China. The institution manages 125 inpatient beds and 18 ICU beds, receives approximately 1,300 burned patients annually (12), and integrates medical treatment, rehabilitation, nursing, and scientific research of burns. The study was approved by the Ethics Committee of the First Affiliated Hospital of Third Military Medical University of PLA (KY201817).

### Study Population and Data Collection

Clinical, biological and anamnestic data on each patient received from January 2011 to January 2017 were collected. Criteria of enrollment in the study were age ≥18 years and burn area ≥30% of total body surface area. Patients living up to the following criteria were excluded: aged under 18 years, or with identified chronic kidney disease (CKD) before the injury, or without detailed prehospital information, or those missing loss to follow-up.

Data were retrospectively collected from clinical database and patients’ electronic medical records in Southwest Hospital. The demographic data included gender, age, body mass index (BMI), cigarette smoking, hypertension, and diabetes. The injury-related data included TBSA, full thickness of TBSA, abbreviated burn severity index score (ABSI) (13), inhalation injury, hypovolemic shock of early burn, decompression escharotomy of circumferential burns, tracheotomy, and etiology of burn (thermal, electric and chemical). Clinical intervention data included: surgeries 1 week prior to AKI, hypotension prior to AKI, max serum myohemoglobin prior to AKI, and sepsis. Clinical outcome data included: 28 and 90 d mortality, development of sepsis and septic shock, use of vasopressors (dopamine, dobutamine, noradrenaline, adrenaline, etc.), length of mechanical ventilation, use of RRT, and intensive care length of stay (ICU-LOS).

APACHE II (14), SOFA (15), and some laboratory values when AKI was diagnosed were also collected, such as: the serum creatinine (sCr), blood urea nitrogen (BUN), cystatin C, serum myohemoglobin, mean hourly urine output, and arterial blood gas analysis including pH, oxygenation index (PaO_2_/FiO_2_) and HCO_3_^-^. Occurrence time and duration of AKI were recorded as well.

### Definition

AKI diagnoses were made according to the KDIGO classification. KDIGO criteria for the diagnosis of AKI include (16): an absolute increase in sCr of ≥0.3 mg/dL (≥26.5 µmol/L) within 48 h, or an increase in sCr 1.5 times the baseline value (or first measurement), or UOP <0.5 mL/kg/h for more than 6 h. Baseline sCr was defined as the lowest value within hospitalization (17). The area of burn wound was assessed by “Chinese rule of nine”, and the burn depth by the “Three degree and four categories” (12).

Sepsis and septic shock were defined according to the “International Guidelines for Management of Sepsis and Septic Shock: 2016” (18). Septic shock was defined as sepsis-induced hypotension persisting with a systolic blood pressure (SBP) <90 mmHg or a mean arterial pressure (MAP) <70 mmHg or a SBP decrease >40 mmHg) despite adequate fluid resuscitation (18).

### Statistical Analysis

The quantitative data were expressed as mean (SD) or median (interquartile ranges, IQR) according to their distributions, while categorical data were expressed as counts (proportions). Then we created a binary outcome variable for AKI: a value of 2 without AKI, 0 when there was early AKI (0–3 days post burn), or value of 1 when there was late AKI; also, binary outcome variables for mortality, and other clinical status. This relied on the fact that early AKI mostly occurs within the 3 days after severe burn, which renders the predictive model for AKI (1). All statistical analyses were performed using SPSS (Version 25; IBM, Armonk, NY, USA).

The Student’s t-test was applied if a normal distribution was detected, while the Mann-Whitney *U*-test was applied for non-normal distributions. Wilcoxon rank sum test or Pearson χ^2^ test for nonparametric variables was used when appropriate.

Risk factors in early AKI and non-AKI, late AKI and non-AKI groups were evaluated in univariate analysis first. Then multivariate logistic regression was applied to the relevant variables from the clinical/physiological perspective. The odd ratios (OR) and 95% confidence intervals (CI) were estimated.

Moreover, ROC curves were built for serum myohemoglobin associated with AKI when the AUC-ROC was reported. Meanwhile, the sensitivity, specificity, positive and negative predictive values, and positive and negative likelihood ratios (PPV, NPV, PLR and NLR) were calculated for serum myohemoglobin. The Youden index (sensitivity + specificity−1) was applied to defining the best threshold.

To assess the association between AKI and subsequent mortality, Kaplan-Meier estimate of survival was constructed to compare 90-day survival between each group via stratified log-rank test. Mortality predictors of patients in early AKI and late AKI groups were assessed using multivariable Cox proportional hazards models. Hazard ratios (HR) with corresponding 95% confidence intervals (CI) were calculated. All testing was two-tailed, and statistical differences were considered significant if *p* < 0.05.

### Sample Size Calculation

A previous study on building risk prediction models demonstrated that the calculation of a cohort sample size would precisely provide the required sample size (19). However, the AKI prevalence in the institute database has not been estimated before. Given the reported prevalence of AKI cohort percentages of severely burned patients ranging from 1 to 36% (1), all patients available in the database over the study period (648 severely burned patients) were included to robustly estimates even when AKI prevalence was as low as 3%.

## Results

### General Characteristics and Incidence of AKI

**Figure 1:**
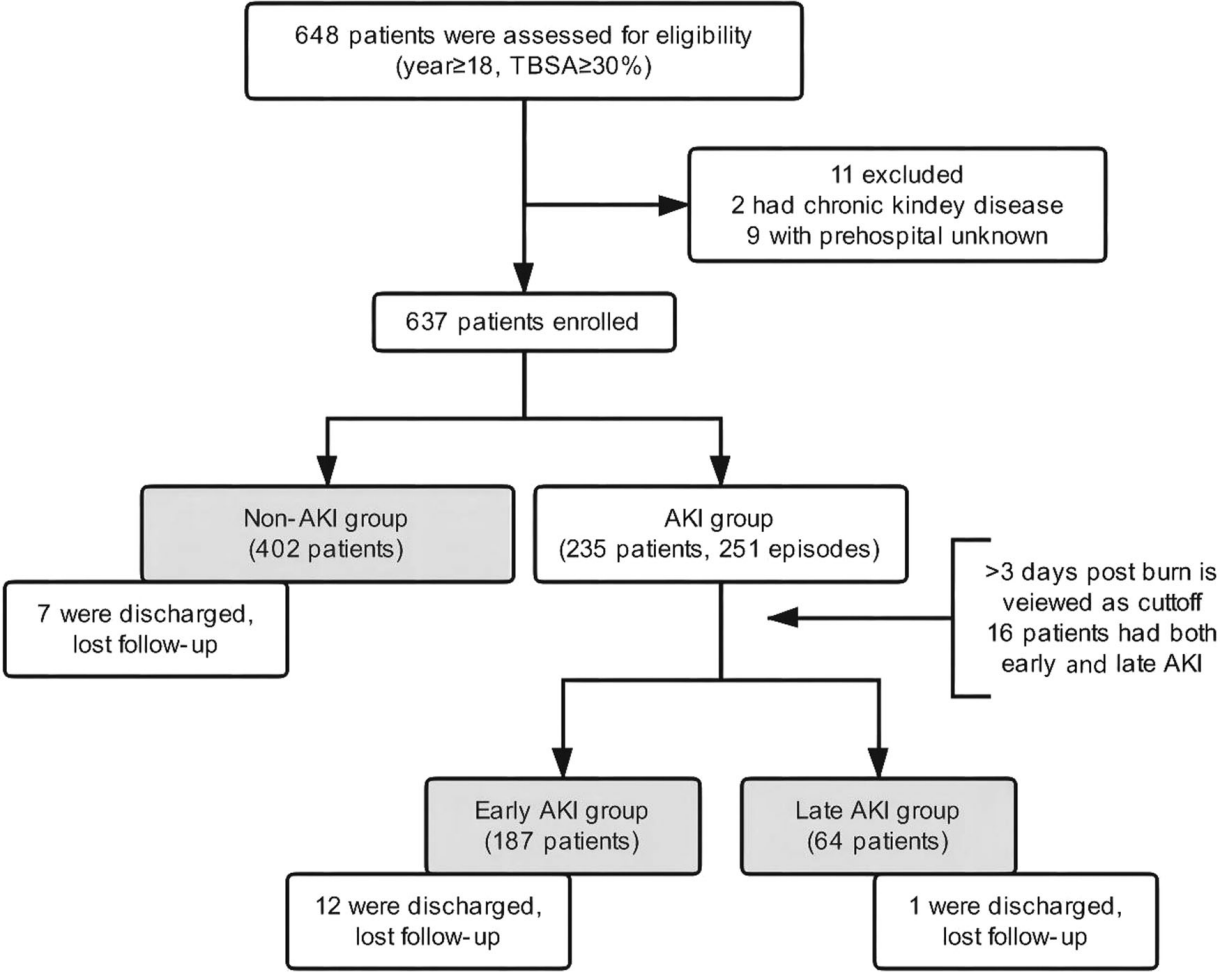
Patient flow diagram. TBSA Total burn surface area, AKI Acute kidney injury.

From the population of all adult burn patients (17,972 people), 648 patients were severely burned and enrolled, from whom 9 patients were excluded because of unspecified prehospital information and 2 patients because of CKD before burns. There were 20 patients discharged without a cure and missing to follow-up (early AKI 12, late AKI 1, and non-AKI 7). To avoid exclusion of a large proportion of patients, these 20 patients underwent analysis of risk factors but were excluded from the analysis of clinical outcomes. At last, 637 patients including 402 non-AKI patients and 235 AKI patients were included into the analysis for our study. Of them, 16 patients developed both early and late AKI, and were recorded in both early and late groups. Thus, the AKI group contained 251 events (**Figure 1**). The incidence of AKI was 36.9% (early AKI 29.4%, late AKI 10.0%). Characteristics of the patients are presented in **Table 1**. Most patients were middle-aged (45 ± 13.2) and male (499, 76.1%), and 201 (30.1%) patients developed hypovolemic shock of early burn. The median value for TBSA, full-thickness burns of TBSA, and ABSI was 45.7% TBSA, 15.4% TBSA, and 9.9, respectively. The two patients with preinjury CKD (stage 3) had developed early AKI after burns, one had given up treatment and discharged (died during follow-up), and the other one developed End-stage renal disease (stage 5) with maintenance hemodialysis.

**Table 1:** Characteristics of the patients.

	Whole cohort (*n* = 637)	Early AKI^a^ (*n* = 187)	Late AKI^a^ (*n* = 64)	Non- AKI (*n* = 402)	*p* value
Age, $\bar{x}\pm s$	45 ± 13.21	47.35 ± 15.27^†^	47.62 ± 11.87^†^	43.58 ± 12.78	0.002
Male, *n* (%)	499 (76.4)	158 (84.9)^†^	59 (92.2)^†^	282 (70.1)	<0.001
BMI, $\bar{x}\pm s$	23.31 ± 3.11	23.20 ± 3.31	23.35 ± 3.65	23.16 ± 3.58	0.918
Smoke, *n* (%)	215 (32.9)	57 (30.6)^‡^	31 (48.4)^†^	127 (31.6)	0.021
Drink, *n* (%)	52 (7.9)	14 (7.5)	10 (15.6)	28 (7.0)	0.057
Hypertension or/and Diabetes, *n* (%)	26 (3.9)	16 (8.6)^†^	5 (7.8)^†^	5 (1.2)	<0.001
Hypotension, *n* (%)	54 (8.3)	11 (5.9)^‡^	30 (46.9)^†^	13 (3.2)	<0.001
TBSA, Median (IQR)	45.7 (33.0)	59.5 (36.0)^‡^	75.0(32.0)^†^	40.5 (20.0)	<0.001
Full-thickness burns of TBSA, Median (IQR)	15.4 (30.0)	29.0 (31.0)^†^	28.5 (31.0)^†^	7.0 (21.0)	<0.001
ABSI, Median (IQR)	9.9 (3.0)	11.0 (3.0)^†^	12.0 (2.0)^†^	9.0 (3.0)	<0.001
Etiology of burn, *n* (%)					0.230
Thermal	616 (94.3)	162 (86.6)	60 (93.8)	349 (86.8)	
Electric	61 (9.3)	20 (10.8)	1 (1.6)	40 (10.0)	
Chemical	21 (3.2)	5 (2.7)	3 (4.7)	13 (3.2)	
Inhalation injury, *n* (%)	207 (31.7)	13 (60.8)^†^	40 (62.5)^†^	154 (38.3)	<0.001
Severity of inhalation injury					0.043
Mild, *n* (%)	154 (23.6)	49 (43,4)	18 (45.0)	87 (56.5)	
Moderate, *n* (%)	102 (15.6)	44 (38.9)	12 (30.0)	50 (32.5)	
Severe, *n* (%)	47 (7.2)	20 (17.7)	10 (25.0)	17 (11.0)	
Hypovolemic shock of early burn, *n* (%)	201 (30.1)	73 (39.2)^†,^^‡^	48 (75.0)^†^	80 (19.9)	<0.001
Tracheotomy, *n* (%)	285 (43.6)	121 (65.1)^†^	48 (75.0)^†^	116 (28.9)	<0.001
Decompression escharotomy, *n* (%)	156 (23.9)	57 (30.6)	12 (18.8)	87 (21.6)	0.035
Surgeries in 1-week prior AKI, *n* (%)	327 (50)	6 (3.2)^†,^^‡^	27 (42.2)^†^	294 (73.1)	<0.001
Sepsis, *n* (%)	62 (9.5)	4 (2.2)^‡^	40 (62.5)^†^	18 (4.5)	<0.001

^†^ *Compared with non- AKI group, p < 0.05.*

*^‡^* *Compared with Late AKI group, <0.05.*

*^a^* *16 patients had both early and late AKI.*

The baseline values of clinical parameters at onset of AKI are summarized in Additional File S1. The average time of initial diagnosis of early AKI was 19 h (the earliest time, 3 h) after burn, while 19 days in late AKI group. Half of the patients with late AKI were unable to restore kidney function, accounting for a significantly higher proportion than those with early AKI (18.19%, *p* < 0.0001). For patients with renal function recovery, the mean AKI duration was 4.2 days in early AKI patients, which was significantly shorter than that in late AKI patients (6.5 days). Incidence of late AKI was lower than that of early AKI, but late AKI patients had more severity of illness and worse prognosis. On the day of AKI diagnosis, patients with late AKI had a higher illness severity, APACHEII score, SOFA score, bicarbonate radical, serum creatinine, BUN and cystatin C levels, than those with early AKI (21.0 vs. 15.0, 8.0 vs. 3.0, 21.4 vs. 17.7, 156.0 vs. 125.0, 15.3 vs. 8.5, and 1.7 vs. 0.8, respectively, all *p* values <0.05). All patients with late AKI were admitted into ICU of burn, while 30 patients with early AKI were not. Moreover, the mean hourly urine output of patients with late AKI (100 mL/h) did not decrease compared to normal adult urine output, while it decreased sharply in early AKI (40 mL/h, *p* < 0.001); pH, PaO_2_/FiO_2_ and lactate in arterial blood had no significant differences between the two groups.

Furthermore, the prevalence showed that a correlation existed between AKI and age or TBSA (**Table 2**). We noted that as the TBSA and age of the patient increased, the incidence of AKI did as well. Specifically, 79% of the patients with burn >80% TBSA developed AKI, and over half of the patients aged above 60 years developed AKI (51.4%).

**Table 2:** Prevalence of AKI in the overall population and in subgroups.^a^

	Non- AKI	Early AKI	Late AKI	*p* value
Overall population(*n* = 637), *n*%	402 (63.1)	187 (29.4)^†^^,^^‡^	64 (10.0)	<0.001
Age (years), *n*%				
18–40	157 (67.4)	59 (25.3)^†,‡^	17 (7.3)	<0.001
40–60	210 (60.3)	98 (28.2)^†^	40 (11.5)	0.046
Over 60	35 (48.6)	30 (41.7) ^‡^	7 (9.7)	0.037
TBSA (%), *n*%				
30–50	282 (78.3)	63 (17.5)^†,‡^	15 (4.2)	<0.001
50–80	99 (51.3)	69 (35.7)^‡^	25 (13.0)	0.013
80–100	21 (21.0)	55 (55.0)^†,‡^	24 (24.0)	0.041

*Data are presented as percentages.*

*^†^* *Compared with Non- AKI group, p < 0.05*

*^‡^* *Compared with Late AKI group, <0.05.*

*^a^* *16 patients had both early and late AKI.*

### Multivariate Analysis of Risk Factors

To eliminate the influence of confounding factors, we analyzed the results by unordered multinomial logistic regression with the variables with *p* < 0.1 from **Table 1**, and the regression results are presented in **Table 3**. The following factors were associated with AKI and considered as independent risk factors associated with early and late AKI: male, age, TBSA, full-thickness burns of TBSA, hypertension or/and diabetes, hypovolemic shock of early burn, and tracheotomy. Sepsis had the greatest influence on late AKI occurrence (OR = 12.83 (5.01–32.85), followed by hypovolemic shock of early burn (OR = 10.23 (4.93–21.25). However, decompression escharotomy was a protective factor for early AKI (OR = 0.45 (0.26–0.78) and late AKI [OR = 0.19 (1.58–8.72)], which reduced the risk by nearly 55% and 81%, respectively. The AUC-ROC of the model was 0.812 (0.738–0.831) for early AKI and 0.833 (0.818–0.853) for late AKI.

**Table 3:** Multinomial logistic regression analysis of associated factors in early AKI and late AKI.

Covariates associated with AKI	Early AKI group		Late AKI group	
	OR (95%CI)	*p* value	OR (95%CI)	*p* value
Age	1.043 (1.019–1.067)	<0.001	1.056 (1.018–1.096)	0.004
Male	3.489 (1.771–6.874)	<0.001	5.055 (1.413–18.079)	0.013
TBSA	1.043 (1.006–1.081)	0.021	1.081 (1.018–1.148)	0.011
Full-thickness burns of TBSA	1.036 (1.021–1.050)	<0.001	1.028 (1.009–1.048)	0.004
ABSI	0.798 (0.567–1.122)	0.194	0.674 (0.376–1.210)	0.186
Hypertension or/and Diabetes	6.739 (2.054–22.112)	0.002	7.846 (1.586–38.809)	0.012
Smoke	0.629 (0.384–1.029)	0.065	1.111 (0.540–2.288)	0.775
Drink	0.905 (0.401–2.043)	0.81	1.483 (0.526–4.183)	0.456
Inhalation injury	1.389 (0.787–2.450)	0.257	1.023 (0.415–2.522)	0.96
Hypovolemic shock of early burn	2.044 (1.267–3.296)	0.003	10.232 (4.926–21.249)	<0.001
Tracheotomy	2.335 (1.344–4.055)	0.003	3.708 (1.577–8.717)	0.003
Decompression escharotomy	0.452 (0.262–0.781)	0.004	0.189 (0.078–0.455)	<0.001
Sepsis	0.115 (0.033–0.404)	0.001	12.831 (5.011–32.854)	<0.001

*Risk factors associated with the occurrence of early and late AKI in a multinomial logistic regression model. Results are presented as Odds Ratio (OR) and 95% confidence interval (CI).*

### Serum Myohemoglobin and Prediction of AKI for Severe Burn Patients

**Figure 2:**
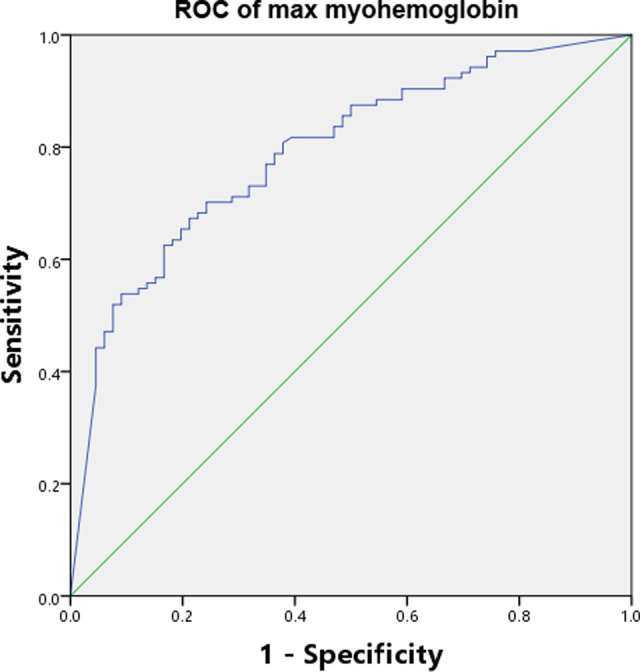
AUC-ROC of serum myohemoglobin. AUC-ROC Area under the curve-receiver operating characteristic curve.

The discriminating power of serum myohemoglobin had an AUC-ROC of 0.79 (0.75–0.82) to predict all AKI (**Figure 2**). Results of additional analyses, including sensitivity, specificity, PPV, NPV, PLR, NLR and optimal cutoff, are available in Additional File S2.

### Outcomes

**Figure 3:**
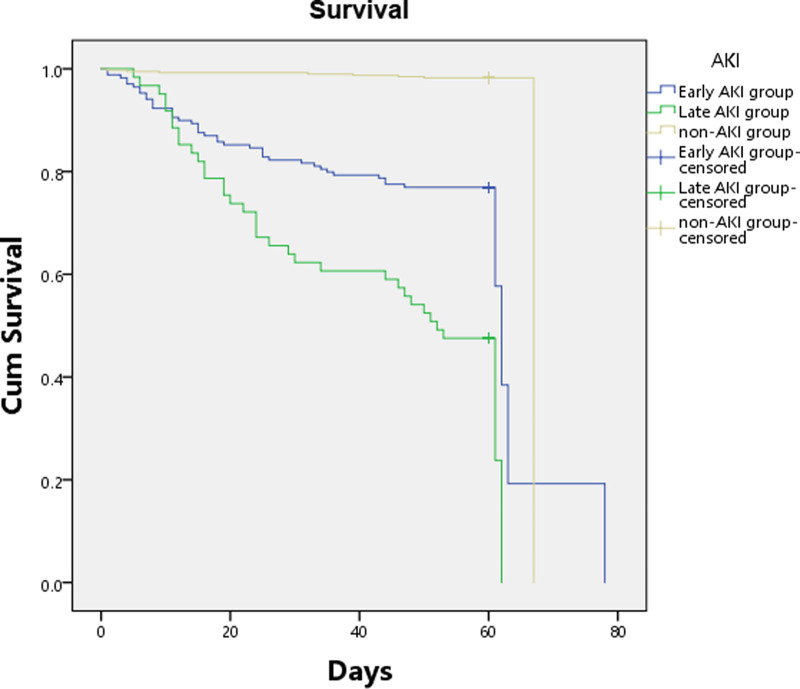
Kaplan-Meier estimate of 90-day survival between each group.

In the 235 burned patients with AKI, 13 patients were not included in the outcome analysis for loss of follow-up (early AKI 12, late AKI 1, Additional File S3). Seventy-six of 222AKI patients (34.2%) died [early AKI 27.4% (48/175), late AKI 57.1% (36/63)], and half of 16 patients with both early and late AKI were died. The survival of severely burned patients was influenced by AKI, the Kaplan-Meier curve demonstrated significant differences in 90-day survival between groups on the basis of the log-rank test (**Figure 3**). None of the patients died more than 90 days after follow-up. Compared with patients without AKI, patients with AKI had significantly higher 28-day mortality [23.0% (51/222) vs. 1.3% (5/395), *p* < 0.001], 90-day mortality [34.2% (76/222) vs. 2.53% (10/395), *p* < 0.001], and worse other outcomes including incidence of sepsis (41.4% vs. 4.6%, *p* < 0.001) and septic shock (23.5% vs. 2.0%, *p* < 0.001), duration of mechanical ventilation (8.5 vs. 4 days, *p* < 0.01), length of ICU stay (25 vs. 10 days, *p* < 0.0001), requirements of vasopressors (39% vs. 6.3%, *p* < 0.001) and RRT (29% vs. 1.8%, *p* < 0.0001) (Additional File S3). Compared with patients with early AKI, patients with late AKI had higher 28-day mortality [34.9% (22/63) vs. 19.4% (34/175), *p* = 0.007], 90-day mortality [57.1% (36/63) vs. 27.4% (48/175), *p* < 0.0001], and higher incidence of sepsis (74.2% vs. 32.6%, *p* < 0.001) and septic shock (55.6% vs. 13,7%, *p* < 0.001) (Additional File S3). To predict the outcome of the severely burned patients, multivariable Cox proportional hazards models in Additional File S3 demonstrated that AKI was independently associated with obviously increase in mortality of severely burned patients [early AKI, OR = 12.98 (6.08–27.72); late AKI, OR = 34.02 (15.69–73.75)].

## Discussion

In this retrospective observational study, it was found that firstly, AKI was still a serious issue in severely burned patients with 36.9% occurrence, and AKI was independently associated with a threefold risk of mortality in severely burned patients. Second, it was obvious that AKI occurred early, with 74% of AKI diagnosed within the first 3 days after severe burn injuries, but late AKI had higher mortality. Third, the AKI predicting model was proved to include age, gender, TBSA, full-thickness burns of TBSA, chronic comorbidities (hypertension or/and diabetes), hypovolemic shock of early burns, inhalation injuries, and serum myohemoglobin. Tracheotomy and sepsis were independent risk factors for AKI.

AKI is a common complication in severely burned patients. The incidence of AKI in burned patients varied from 1% to 36% depending on the different population studied and diagnostic criteria, and the mortality among burned patients with AKI was 28% to 100% (20). Other studies have shown that AKI occurred in 53.3% of patients with severe burn injuries, and patients with AKI had a mortality rate of 34.4% (4). In this study, the incidence of AKI was 36.9% in the patients with burn ≥30% TBSA; the total mortality was 34.2% in the patients with AKI, highest in the patients with late AKI with the mortality up to 57.1%, which was higher in burn patients than in general population (5). Therefore, it is crucial to early prevent and diagnose AKI in severely burned patients. However, in our other study, we found the incidence and mortality of AKI have not decreased in the consecutive six years. It is particularly urgent to reduce occurrence of AKI after burn.

In multinomial logistic regression, the study found that age, gender (male), TBSA, full thickness burns of TBSA, hypertension or/and diabetes, tracheotomy and hypovolemic shock of early burns were independently associated with both early and late AKI development, and sepsis was an independent risk factor for late AKI. However, BMI and drink, smoke, ABSI, etiology of burns and inhalation injuries had no influences, which is similar to previous studies (21–23). The risk of the aged and male patients suffering early and late AKI increased by over one to five folds, which is in agreement with some studies (24–26), while others found gender is not a predisposing factor for AKI (10). Considering that more male patients may be engaged in electrical works and suffered electrical injuries which may be a risk factor for AKI, this result was not changed after analyzing the cases of thermal burns (data not shown). The severity of burns is largely determined by burn area and depth. Major burn size is associated with multiple organ dysfunction and poor prognosis, and burn size is an independent predictor of acute renal failure (13). In this study, TBSA and full-thickness burns of TBSA strongly correlated with AKI. Patients with hypertension or/and diabetes underwent chronic pathological changes in organs including the heart, brain, kidneys and blood vessels causing deterioration of renal function, and were more predisposed to AKI (20). Inhalation injury was a severe complication of burn injuries, and was often associated with AKI (23). Though univariate analysis in this study showed significant difference in inhalation injury between the groups, there was no evidence of an independent inhalation injury effect after adjusting for potential confounders. This result is consistent with recent study (27). This could be because most mild to moderate inhalation injuries in this study did not develop to ARDS, which was proved to be a risk factor for AKI. ARDS is an important pathophysiologic link between acute lung injury and AKI and the existence of the “lung-kidney crosstalk” (27–29). Tracheotomy was an independent risk factor for early AKI [OR = 2.34 (1.34–4.01)] and late AKI [OR = 3.71(1.58–8.72)]. This can be explained as that the patients with tracheotomy were more seriously ill with inadequate oxygenation and needed mechanical ventilation support. Meanwhile, there may be complications of secondary pulmonary infection induced by tracheotomy and mechanical ventilation. However, we also included a few patients to perform prophylactic tracheotomy for compression of the neck and laryngeal edema after burns.

There are different pathophysiological features and potential mechanisms for burn-related AKI at different stages after burns. Hypovolemic shock is the main complication of major deep burns in the resuscitative phase, with a hugely harmful influence on the whole course of severe burns (30), when consequent sepsis and MODS increased the mortality accordingly. This study confirmed that hypovolemic shock of early burns is also a risk factor for AKI in severely burned patients. It has always been believed that renal hypoperfusion leads to ischemia and ischemia-reperfusion injury, and timely and aggressive fluid resuscitation can reduce the incidence of AKI and improve prognosis (31). We paid attention to the early fluid resuscitation after burns, and drafted the TMMU protocol for fluid resuscitation in the 1960s, which has been followed to save lives of innumerable burn patients, and until now it is still widely used in China (32). However, aggressive fluid resuscitation did not completely avoid AKI (33, 34). Furthermore, excess resuscitation was associated with some complications, including pneumonia, ARDS and elevated compartment syndromes (33, 35). This study found the earliest onset of AKI was 3 h (or may be earlier) after burns, and AKI was still developed in burn patients who received rapid fluid resuscitation, with normal or slight decrease of urine output. Hemorrhagic shock and septic shock in animal experiments demonstrated that immediate fluid resuscitation was sufficient to restore systemic blood pressure but failed to restore renal tissue oxygenation (36, 37). Therefore, etiology of early AKI is multifactorial, not solely focusing on the amount of fluid received. Renal vasoconstriction by stress-related hormones (catecholamines, angiotensin II, aldosterone, vasopression, etc.), inflammatory mediators, denatured proteins (myoglobin, myoglobinuria, free haemoglobinuria, etc.) are probably also associated with the occurrence of AKI (1). It was also observed that sepsis was the main reason for late AKI 3 days after burns, and was an independent risk factor for late AKI. This was consistent with a former study (36). Interestingly, late AKI occurred before the change of urine volume. On the day of initial diagnosis, urine of patients with late AKI did not decrease (100 mL/h), but sCr increased (156 µmol/L). It is likely that renal blood flow did not decrease, but the creatinine clearance has already been markedly reduced. This confirms that the inflammatory mediators and microcirculation dysfunction mainly caused by sepsis and infection contribute to the development of late AKI (38).

Myohemoglobin (myoglobin) is a harmful product of rhabdomyolysis, which is a clinical syndrome secondary to skeletal muscle injury (39). Rapid release of myohemoglobin is the main cause of renal failure, and the pathophysiology is as follows: renal vasoconstriction with resultant ischemia, tubular obstruction due to myohemoglobin cast formation, and direct nephrotoxicity. In severely burned patients, myohemoglobin was proved to have a very stable predictive value for AKI with pronounced sensitivity and specificity (40), as is also reported in other traumatic injuries (41).

It is interesting to note that decompression escharotomy of circumferential burns probably reduced the risk of AKI development. Circumferential eschar, generally resulted from third-degree burns, can cause compression of the underlying soft tissues as burn edema develops and lead to compartment syndrome (42). With the increase of the compartment pressure, ischemia of the underlying tissues and the distal tissues will result in tissue and muscle necrosis, and lactic acidosis without timely decompression escharotomy. Toxins released from necrotic tissue are an important etiology of AKI. Decompression escharotomy should be considered earlier, especially in patients with very deep thermal and/or electrical injuries, as it may be effective in the prevention of AKI.

Several studies have emphasized that AKI is independently related to adverse clinical outcomes in trauma patients (5) and ICU patients (43). In this cohort, AKI was independently associated with high mortality (28- and 90-day mortality). Thus, renal dysfunction serves as an additional predictor of huge risk of mortality in severely burned patients. Mortality of patients with late AKI was significantly higher than those with early AKI as well as incidence of sepsis, septic shock, and usage of vasopressors. Probably main reason of late AKI was sepsis, which had bad prognosis.

This study is not a multicenter study, though the total number of AKI patients in the sample far exceeded the recommended number for building a model to provide predictions (19). This study evaluated comprehensive characterization of clinical parameters of severely burned patients with AKI, and demonstrated an association between risk factors and AKI occurrence, but has not established causality. Fortunately, the single center study performs a better stability in burn patients’ data managing, diagnosis criteria and treatment, which ensure that the data are more convincible than a multicenter database where subjective factors might be amplified.

A sensitive and specific biomarker for an accurate early diagnosis of AKI is urgently needed even in the absence of subsequent dysfunction. The novel biomarker is probably a produced material of damaged kidney itself, just like the specific cardiac troponin protein produced immediately when myocardial infarction occurs.

## Conclusions

AKI which is independently associated with high mortality in severely burned patients, is still highly prevalent. Age, gender, TBSA, full-thickness burns of TBSA, hypertension or/and diabetes, tracheotomy, hypovolemic shock of early burns and sepsis are independent risk factors for AKI. Decompression escharotomy is clearly associated with decreased risk for AKI, especially for late AKI. It is crucial to adopt early effective and individualized prevention strategies for severely burned patients to prevent AKI from taking their lives.

## Data Availability

The datasets presented in this study can be found in online repositories. The names of the repository/repositories and accession number(s) can be found in the article/**Supplementary Material**.
