# Supplementary material for: Late-Onset Acute Kidney Injury is a Poor Prognostic Sign for Severe Burn Patients
Source: Front Surg. 2022 May 2;9:842999. doi: 10.3389/fsurg.2022.842999 (PMC9108380; doi:10.3389/fsurg.2022.842999)
Supplement: Supplementary file 1 [file Table_1_v1.pdf]

Supplementary Table 1. Baseline clinical parameters at onset time of AKI

	Early AKI group (n=187)	Late AKI group (n=64)	*P* value
Onset time of AKI	19.13 hours	19 days	-
Duration of AKI (days)	4.2 (3.19) ‡	6.5 (3.28)	<0.001
No recovery of AKI, n (%)	34 (18.19) ‡	32 (50)	<0.001
Mean hourly UOP (ml/h）	40 (39.25) ‡	100 (55)	<0.001
APACHE II	15.0 (5.3) ‡	21.0 (18.0)	0.046
SOFA	3.0 (3.0) ‡	8.0 (4.0)	<0.001
BUN (mmol/L)	8.5 (4.8) ‡	15.3 (20.7)	<0.001
sCr (umol/L)	125.0 (58.5) ‡	156.0 (125.5)	0.508
PH	7.3 (0.2)	7.4 (0.2)	0.002
PaO_2/FiO_2	356.5 (167.0)	352.0 (181.0)	<0.001
Lactate (mmol/L)	3.8 (2.8)	2.95 (5.42)	0.753
HCO_3^- (mmol/L)	17.7 (8.1) ‡	21.4 (11.3)	<0.001
Cystatin C(mg/L)	0.8 (0.7) ‡	1.7 (1.3)	<0.001

Abbreviations: AKI Acute kidney injury, UOP urine output， APACHE II Acute physiology and chronic health evaluation II，SOFA Sequential organ failure assessment, BUN Blood urea nitrogen,,sCr Serum creatinine, PaO_2/FiO_2 Ratio of arterial oxygen partial pressure to the fraction of inspiration oxygen

Data are presented as medians (IQR). All patients were included in comparisons
‡ Compared with late AKI group,p <p0.05
