# Supplementary material for: Late-Onset Acute Kidney Injury is a Poor Prognostic Sign for Severe Burn Patients
Source: Front Surg. 2022 May 2;9:842999. doi: 10.3389/fsurg.2022.842999 (PMC9108380; doi:10.3389/fsurg.2022.842999)
Supplement: Supplementary file 2 [file Table_2_v1.pdf]

Supplementary Table 2. Predictive performances of max myohemoglobin prior AKI

	Cutoff value	Sensitivity	Specificity	Youden index	PPV	NPV	NLR	PLR
Max myohemoglobin prior AKI (ng/ml)	≥364.3	67 (58-77)	79 (74-83)	145.1	31 (27-33)	93 (91-94)	0.54 (0.43-0.61)	2.5(2.1-2.9)
