# Supplementary material for: Late-Onset Acute Kidney Injury is a Poor Prognostic Sign for Severe Burn Patients
Source: Front Surg. 2022 May 2;9:842999. doi: 10.3389/fsurg.2022.842999 (PMC9108380; doi:10.3389/fsurg.2022.842999)
Supplement: Supplementary file 3 [file Table_3_v1.pdf]

Supplementary Table 3a. Outcome of patients involved*

	Early AKI group (n = 187) *	Late AKI group (n = 64) *	Non- AKI group (n = 402) *	*P* value
Lost to follow-up (n)*	12	1	7	-
Sepsis, n (%)	57 (32.6) †‡	47 (74.2) †	17 (4.6)	<0.001
Septic shock, n (%)	24 (13.7) †‡	35 (55.6) †	8 (2.0)	<0.001
ICU length of stay (days)	25 (38) †	25 (16) †	10 (23)	<0.001
28-day mortality, n (%)	34 (19.4) †‡	22 (34.9) †	5 (1.3)	<0.001
90-day mortality, n (%)	48 (27.4) †‡	36 (57.1) †	10 (2.5)	<0.001
Usage of vasopressors, n (%)	54 (30.9) †‡	44 (69.8) †	22 (6.3)	<0.001
Length of Mechanical ventilation (days)	8.5 (9) †	9 (9) †	4 (6)	<0.001
RRT, n (%)	51 (29.1) †	22 (34.9) †	7 (1.8)	<0.001

Abbreviations: ICU Intensive care unit, RRT Renal replacement therapy

Data are presented as medians (IQR) or percentages.

* 20 patients were excluded because of discharge for electing comfort care upon or treatment abandoning. 16 patients of them had both early and late AKI

† Compared with No AKI group, p <0.05: ‡ Compared with late AKI group, p < 0.05

Supplementary Table 3b. Survival analysis (90 days) of the cohort

Groups	Median (95%CI)	HR (95%CI)
Early AKI group	62.0 (60.0-64.0) †‡	12.98 (6.08-27.72)
Late AKI group	52.0 (46.5-57.5) †	34.02 (15.69-73.75)
Non- AKI group	67.0	1.00
All	62.0 (60.7-63.3)	-

† Compared with No AKI group, *p* < 0.05; ‡ Compared with late AKI group, *p* < 0.05
